# Elastin Fiber Accumulation in Liver Correlates with the Development of Hepatocellular Carcinoma

**DOI:** 10.1371/journal.pone.0154558

**Published:** 2016-04-29

**Authors:** Yutaka Yasui, Tokiya Abe, Masayuki Kurosaki, Mayu Higuchi, Yasuyuki Komiyama, Tsubasa Yoshida, Tsuguru Hayashi, Konomi Kuwabara, Kenta Takaura, Natsuko Nakakuki, Hitomi Takada, Nobuharu Tamaki, Shoko Suzuki, Hiroyuki Nakanishi, Kaoru Tsuchiya, Jun Itakura, Yuka Takahashi, Akinori Hashiguchi, Michiie Sakamoto, Namiki Izumi

**Affiliations:** 1 Department of Gastroenterology and Hepatology, Musashino Red Cross Hospital, Tokyo, Japan; 2 Department of Pathology, School of Medicine, Keio University, Tokyo, Japan; National Yang-Ming University, TAIWAN

## Abstract

**Background & Aims:**

The fibrosis stage, which is evaluated by the distribution pattern of collagen fibers, is a major predictor for the development of hepatocellular carcinoma (HCC) for patients with hepatitis C. Meanwhile, the role of elastin fibers has not yet been elucidated. The present study was conducted to determine the significance of quantifying both collagen and elastin fibers.

**Methods:**

We enrolled 189 consecutive patients with hepatitis C and advanced fibrosis. Using Elastica van Gieson-stained whole-slide images of pretreatment liver biopsies, collagen and elastin fibers were evaluated pixel by pixel (0.46 μm/pixel) using an automated computational method. Consequently, fiber amount and cumulative incidences of HCC within 3 years were analyzed.

**Results:**

There was a significant correlation between collagen and elastin fibers, whereas variation in elastin fiber was greater than in collagen fiber. Both collagen fiber (*p* = 0.008) and elastin fiber (*p* < 0.001) were significantly correlated with F stage. In total, 30 patients developed HCC during follow-up. Patients who have higher elastin fiber (*p* = 0.002) in addition to higher collagen fiber (*p* = 0.05) showed significantly higher incidences of HCC. With regard to elastin fiber, this difference remained significant in F3 patients. Furthermore, for patients with a higher collagen fiber amount, higher elastin was a significant predictor for HCC development (*p* = 0.02).

**Conclusions:**

Computational analysis is a novel technique for quantification of fibers with the added value of conventional staging. Elastin fiber is a predictor for the development of HCC independently of collagen fiber and F stage.

## Introduction

For patients with chronic liver disease, the assessment of fibrosis stage is of great importance, because the progression to liver cirrhosis is a major prognostic factor [1]. Liver fibrosis is mainly caused by chronic inflammation because of viral infection, autoimmunity, alcohol consumption, and drug induced liver injury. Viral infection is especially a leading cause of cirrhosis [2]. Cirrhosis is defined as F4 stage in METAVIR staging system, and further fiber accumulation could not sub-classified by conventional staging system. In decompensated cirrhosis, liver-related complications, such as hepatocellular carcinoma, ascites, variceal bleeding, and hepatic encephalopathy, commonly occur. To assess the risk of such complications, multiple noninvasive methods have been studied; however, histological evaluation remains the golden standard for evaluating the stage of fibrosis.

The fibrosis stage is commonly defined by degree and pattern of fiber accumulation. Two types of fibers exist: collagen fibers and elastin fibers. The fibrosis stage is generally diagnosed using sections where the collagen is stained such as Masson’s trichrome stain, but this renders evaluation of elastin fibers challenging. Furthermore, small quantities of elastic fiber make the analysis more difficult. Accordingly, the clinical implications of elastic fiber accumulation remain unknown.

Meanwhile, current computerized histopathology analyses increasingly support measurements of small tissue components [3–7]. According to liver fibrosis, computer-assisted digital analysis such as collagen proportional area method is well established [8–10].

Tsochatzis et al. underwent a study including chronic hepatitis and cirrhosis, and clarified that collagen proportional area significantly correlated the prognosis of these patients. Our previous study also revealed collagen amount in liver tissue correlates the non-invasively measured liver stiffness [11]. In addition, the study also indicated the clinical significance of elastin fiber that was not thought to be worthy of discussion. This results were enabled by novel pathological imaging techniques for whole slide imaging, with which more precise measurements of histological components could be measured [12, 13]. However, this method still needs to be validated and elucidated with regard to its relevance and usefulness in clinical settings.

We aimed to clarify the clinical significance of both collagen and elastin fibers. Because elastin fiber is too small to be evaluated in the patients who were diagnosed METAVIR F1-2, we only included patients with advanced fibrosis. The present study was a retrospective study conducted to determine the significance of quantifying fibers in patients with advanced fibrosis by automated computational analysis using the development of HCC as a clinical parameter.

## Patients and Methods

### Patients

Between January 2003 and March 2011, 1,697 consecutive patients with chronic hepatitis C underwent liver biopsy prior to interferon (IFN) therapy at our referral center. Among these patients, patients fulfilling the following inclusion criteria were included in the present study: Patients had a diagnosis of advanced fibrosis (METAVIR stage F3 or F4) and did not achieve sustained virological response (SVR) with IFN therapy, did not have a past history of HCC, had been followed up at least for one year, and had adequate biopsy sample available for analysis. We collected baseline characteristics and follow-up data. According to the baseline characteristics, APRI (AST to Platelet Ratio Index) [14] and FIB-4 index [15] were calculated using the following formula; APRI = (AST [IU/L]/ upper limit of normal)/platelet [10^9^/L] × 100; FIB-4 = (age [year] × AST [IU/L])/ (platelet [10^9^/L] × √ALT [IU/L]).

Written informed consent was obtained from each patient. The study protocol was approved by the ethics review committees of Musashino Red Cross Hospital and conformed to the ethical guidelines of the Declaration of Helsinki.

### Liver biopsy and histological evaluation

Liver biopsy specimens were obtained using 13 G needles under laparoscopy or using 15 G needles guided by ultrasound. Liver biopsies were considered adequate for histological analyses if they were longer than 8 mm. Liver biopsy specimens were scored by board-certified pathologists, blinded to clinical data, with regard to stage of fibrosis and grade of inflammatory activity according to the METAVIR scoring system.

### Automated quantification of fibers using whole slide imaging

**Fig 1:**
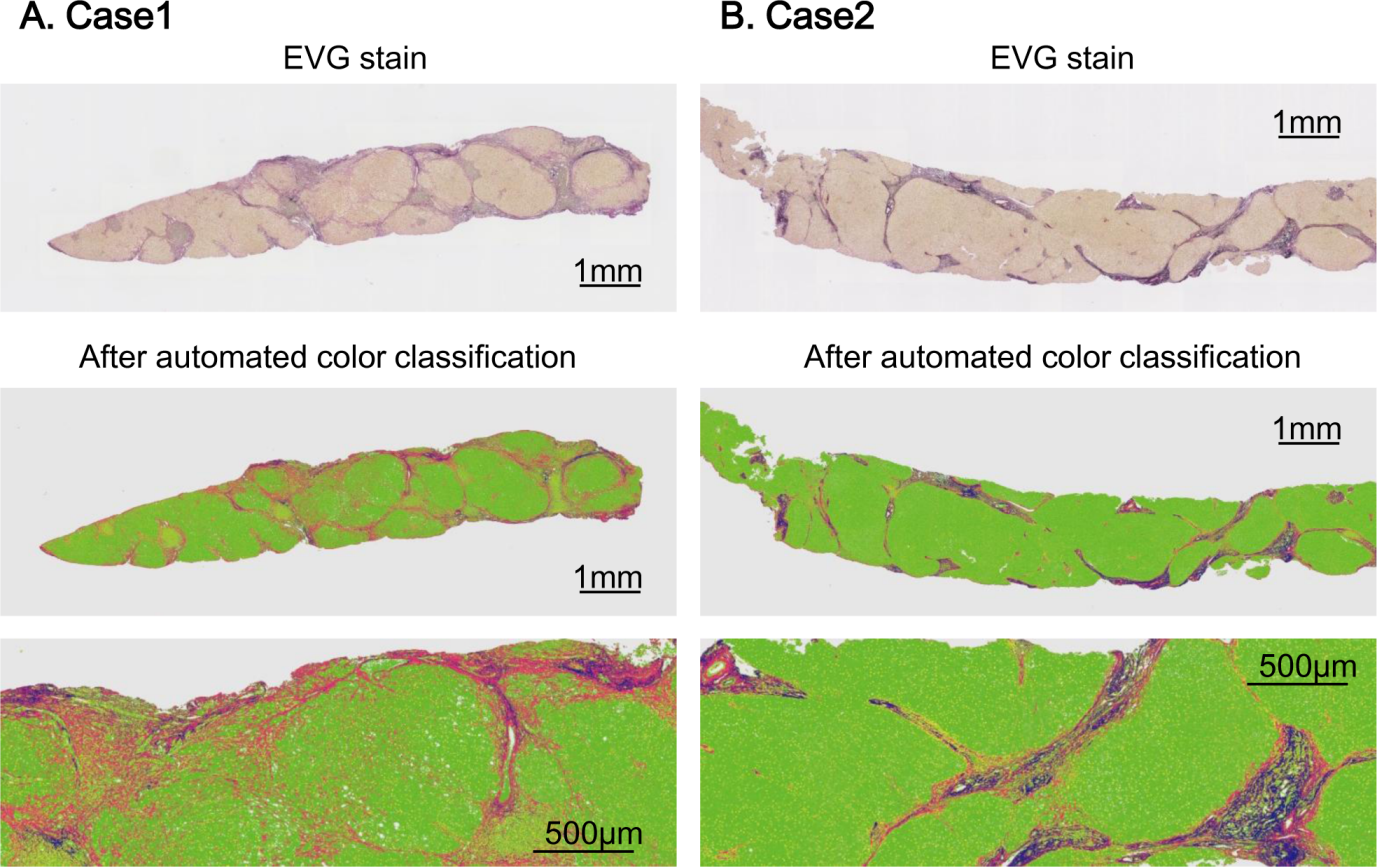
Representative cases of fiber quantification. Panel (A) and (B) show representative cases of automated fiber quantification. Both cases were diagnosed as F4 by METAVIR staging system. After automated color classification, collagen fiber was depicted as red color whereas elastin fiber was depicted as blue color. Case 1 is a patient with relatively low elastin proportional area, who showed 14.1% of collagen and 2.5% of elastin. On the contrary, case 2 is a patient with a higher elastin, who showed 6.5% of collagen and 4.8% of elastin.

In order to precisely measure the accumulation and framework of collagen and elastin fibers, WSI of liver biopsy specimens were analyzed computationally. We used the same method as previously reported [11, 16]. Biopsy specimens were fixed in paraffin blocks and sliced, 4 μm thick. After Elastica van Gieson staining, high-resolution whole slide images (WSI) were generated using the Nano Zoomer 2.0HT (Hamamatsu Photonics K.K., Hamamatsu, Japan) at a × 20 objective lens, equivalent to 0.46 μm/pixel. Calibration of camera was 2173 × 2173 pixels = 4721929 = 1 mm^2^, which is approximately 200 times higher than previously reported [8, 9]. High-resolution WSI enabled accurate, automated quantification of fine collagen and elastin fibers. The WSI were analyzed per pixels with the same method used in the previous studies. All pixels were classified into five classes corresponding to four tissue components: collagen fibers, elastic fibers, nucleus, cytoplasm, and one non-tissue component (i.e., glass slide). According to the analysis of color distributions, all WSI pixels were automatically classified into appropriate components, and a color mapping image was obtained. The area ratio of each tissue component is the sum of pixels for each tissue component divided by the total number of pixels of the four tissue components. The percentages of collagen and elastin fiber areas were calculated as collagen proportional area and elastin proportional area (Fig 1). As shown on the S1 Fig, all processes from acquiring WSI to outputting the median value of fibers are automated. We evaluated fiber amount by calculating piece by piece, compartmenting the biopsy sample by 1 mm^2^ [11] and outputting the median value of fiber amount. Quantified values were expressed as the percentage of each fiber type. To avoid the bias caused by vascular areas, major vascular areas are usually masked and excluded from evaluation. With our method, that bias could be avoided automatically because the median value is not influenced by major vascular areas. In all cases, fibers were adequately quantified by the automated quantification.

### Study end point

The aim of the present study was to assess the relationship between histological fiber quantification and clinical outcome, especially the development of HCC. Patients were examined for HCC every 3–6 months by abdominal ultrasonography, dynamic computed tomography, or magnetic resonance imaging. Serum alpha-fetoprotein levels were measured every 3 months. The diagnosis of HCC was confirmed by needle biopsies, surgically resected specimens, or according to typical radiological hallmarks of early enhancement and delayed washout. The first day of follow-up was the date of the liver biopsy, and the last day of follow-up was the day of establishing the HCC diagnosis or 3 years after liver biopsy.

### Statistical analysis

Continuous variables were reported as means with standard deviations (SD), and categorical variables were shown as frequencies and percentages. Statistical significance was assessed using Student’s t test (mean) or Fisher’s exact test. Cumulative incidences of HCC were analyzed with the Kaplan–Meier method. Two-sided probability (p) values were calculated in all tests, and differences were considered statistically significant when p < 0.05. Cox proportional hazard models were used to explore independent factors that could be used to predict HCC development. Cut off values were calculated by ROC (receiver operating curve) analyses for significant factors. For other factors, cut off value was set as at the upper limit normal or the median of baseline. All variables with *P* values of < 0.1 from the univariate analysis were included in the multivariate analyses using backward elimination method.

Statistical analyses were performed using the Statistical Package for the Social Sciences software version 20.0 (SPSS, Chicago, IL).

## Results

### Patient characteristics

A total of 402 patients were diagnosed with advanced fibrosis. Among these, 110 (27.3%) achieved SVR, 28 (6.9%) had a past history of HCC, 57 (18.4%) did not undergo appropriate follow-up or moved, and 18 (4.5%) were not eligible because of the unavailability of adequate biopsy material. A total of 189 consecutive HCV-infected patients with advanced fibrosis not achieving SVR by IFN therapy were included in the study. Among these 154 patients HCV genotyping have been made, which revealed 128 genotype 1b patients (83%) and 26 non genotype 1b patients (17%). 52 patients (30.2%) were diagnosed with cirrhosis. Median levels of collagen proportional area and elastin proportional area were 9.1% and 2.7%, respectively. Table 1 describes the baseline characteristics of the patients included in the present study.

**Table 1:** Baseline characteristics of 189 chronic hepatitis C patients with advanced fibrosis.

Characteristics	n = 189
Age (SD) in years	63.7 (8.3)
Sex, n (%)	
Male	78 (41)
Female	111 (59)
Biochemistry	
AST (SD), U/L	74 (38)
ALT (SD), U/L	80 (52)
Bilirubin (SD), mg/dL	0.8 (0.3)
Platelet (SD), 10^6^/μL	12.1 (4.1)
Albumin (SD), g/dL	3.8 (0.4)
GGT (SD), IU/L	65 (62)
Glucose (SD), mg/dL	114 (36)
Cholesterol (SD), mg/dL	165 (31)
PT-INR (SD)	1.03 (0.09)
AFP (SD), ng/mL	25.8 (54.3)
HCV genotype, n	
1a/ 1b/ 2a/ 2b/ NA	2/ 130/ 17/ 5/ 35
Histology	
Fibrosis stage, n (%)	
F3	132 (70)
F4	57 (30)
Activity stage, n (%)	
A0/1	30 (16)
A2	129 (68)
A3	30 (16)
Fibers (SD), %	
Collagen proportional area	9.9 (4.0)
Elastin proportional area	3.6 (3.1)

Abbreviations: AST, aspartate aminotransferase; ALT, alanine aminotransferase; GGT, gamma glutamyl transferase; AFP, alpha-fetoprotein; NA, not available

Data are presented as proportions (%) or means (SD).

### Correlation between automatically quantified fiber accumulation and METAVIR stage

**Fig 2:**
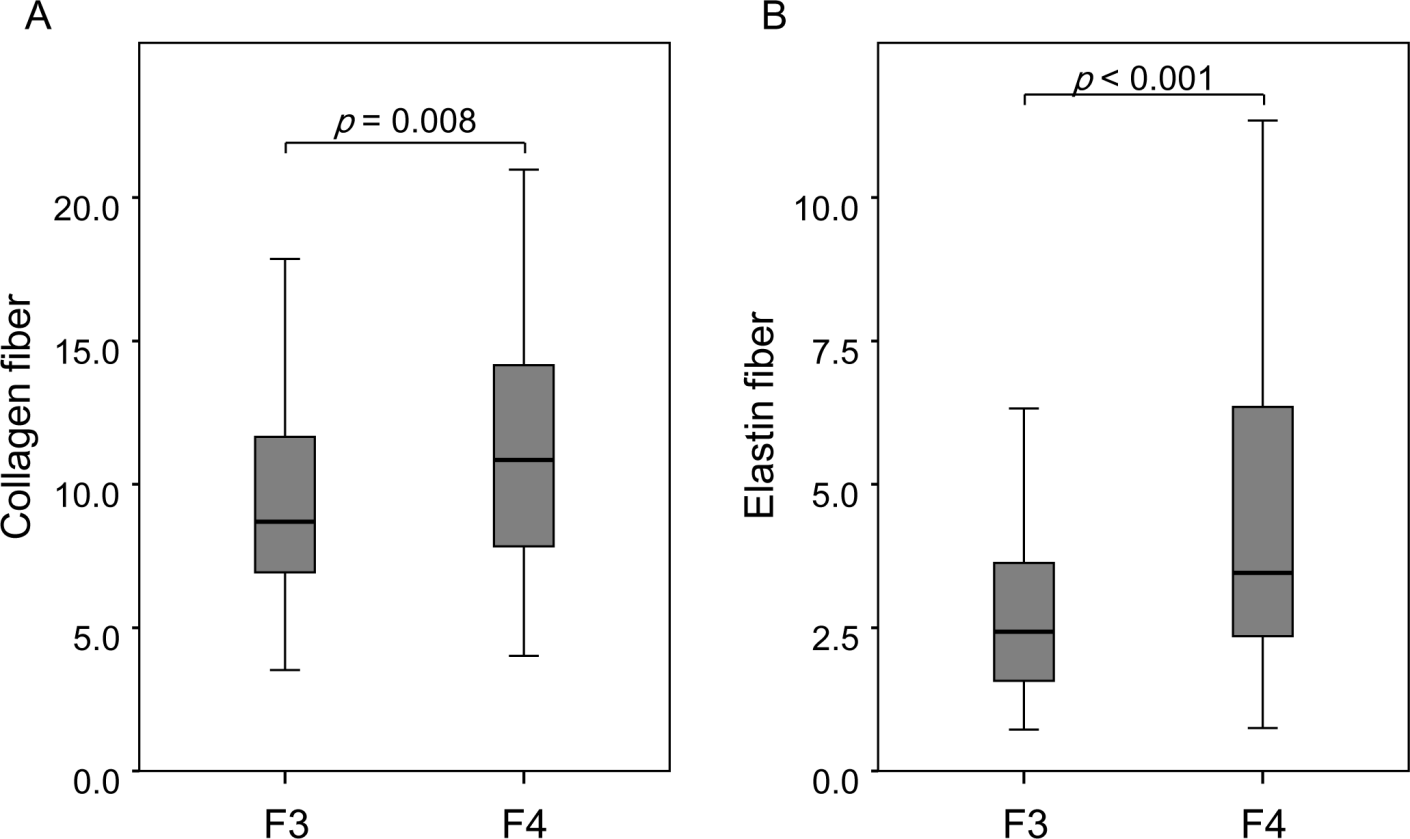
Box plot of fibers content distribution according to F stage. Both collagen fibers (A) and elastin fibers (B) differed significantly between F3 and F4. Gray boxes indicate interquartile ranges, and horizontal lines indicate the median value.

**Fig 3:**
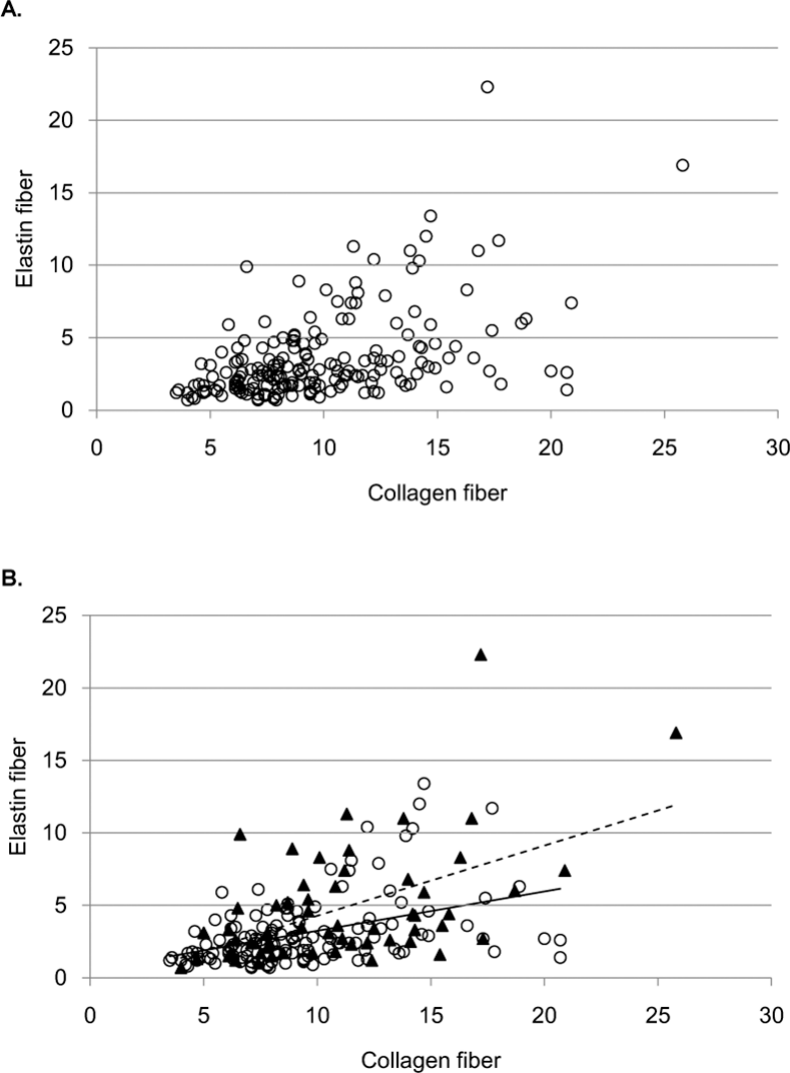
Relationship between collagen fibers and elastin fibers. Panel (A) shows a scatter plot of the significant correlation between collagen and elastin. As the collagen increase, the variance of elastin also increases. Panel (B) shows a scatter plot classified by F stage. A circle indicates an F3 patient, whereas a triangle indicates an F4 patient. The solid line is the trend line for F3 patients, whereas the broken line is the trend line for F4 patients.

Comparing F3 patients (n = 132) with F4 patients (n = 57) indicated that there was significant correlation between fibrosis stage and collagen proportional area [F3, 9.4% vs. F4, 11.1% (mean), *p* = 0.008]. This result was also observed for elastin proportional area [F3, 3.1 vs. F4, 4.8 (mean), *p* < 0.001]. Moreover, the range of elastin fiber contents was wider in patients diagnosed as F4 than those of F3 (Fig 2). Fig 3 shows relationship between collagen fibers and elastin fibers. Although there was a significant correlation between collagen and elastin (*r* = 0.477, *p* < 0.001), the coefficient of variation was higher for elastin than for collagen. In other words, some patients showed high elastin proportional area despite having low collagen proportional area and vice versa. This difference was higher in patients with higher collagen proportional area (Fig 3A). On the other hand, according to activity stage, A0/1, A2, A3 were observed in 30, 129, and 30 patients at baseline. Activity stage was not correlated with elastin fiber accumulation.

### Quantified fiber and cumulative incidence of hepatocellular carcinoma

**Fig 4:**
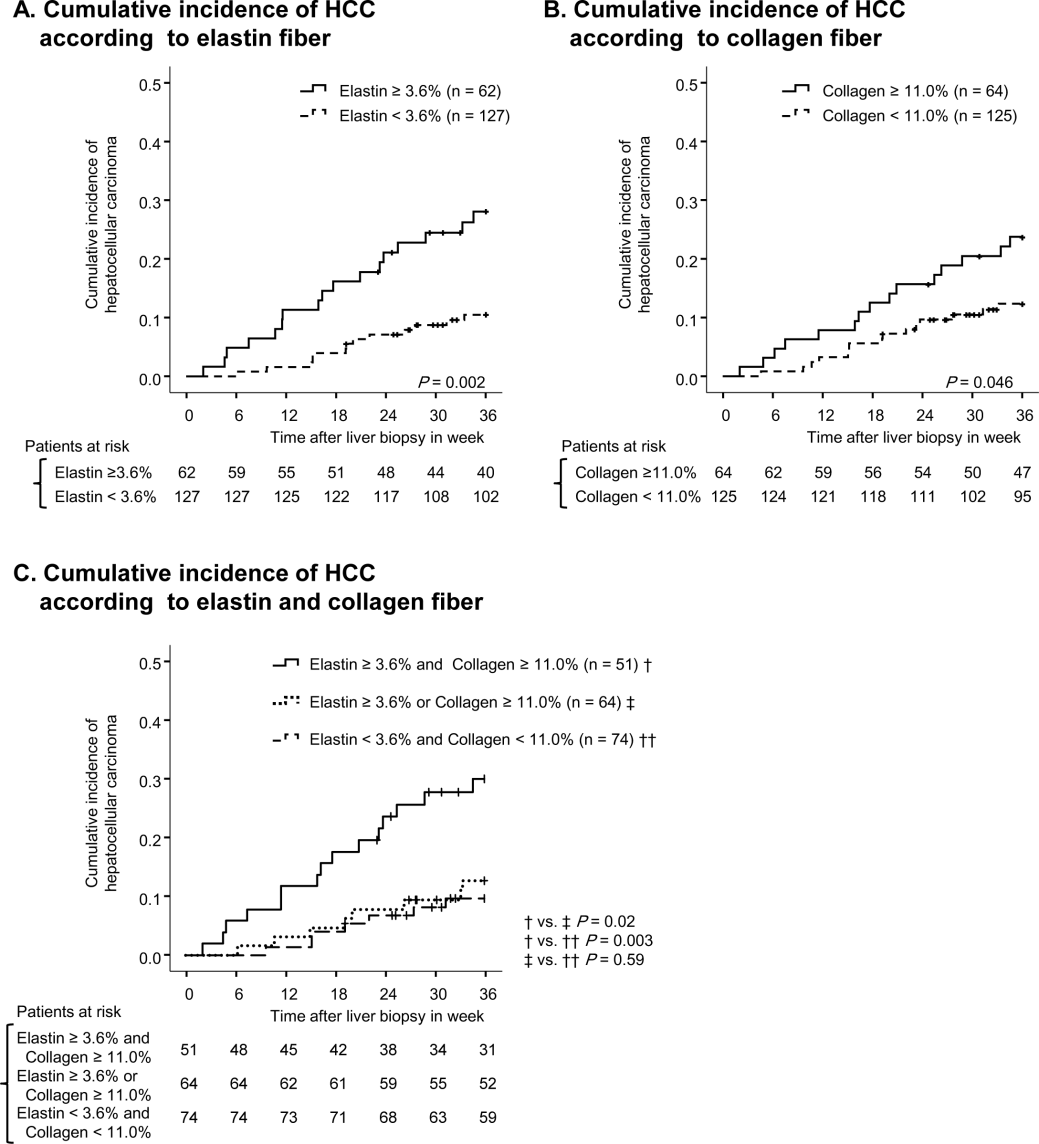
Cumulative incidence of HCC according to fiber content. On panel (A), the cumulative incidence of HCC is compared between patients with high elastin (solid line) and low elastin (broken line). On panel (B), the cumulative incidence of HCC is compared between patients with high collagen (solid line) and low collagen (broken line). Vertical lines indicate censored cases. On panel (C), the cumulative incidence of HCC is compared in three groups divided by collagen and elastin content.

**Fig 5:**
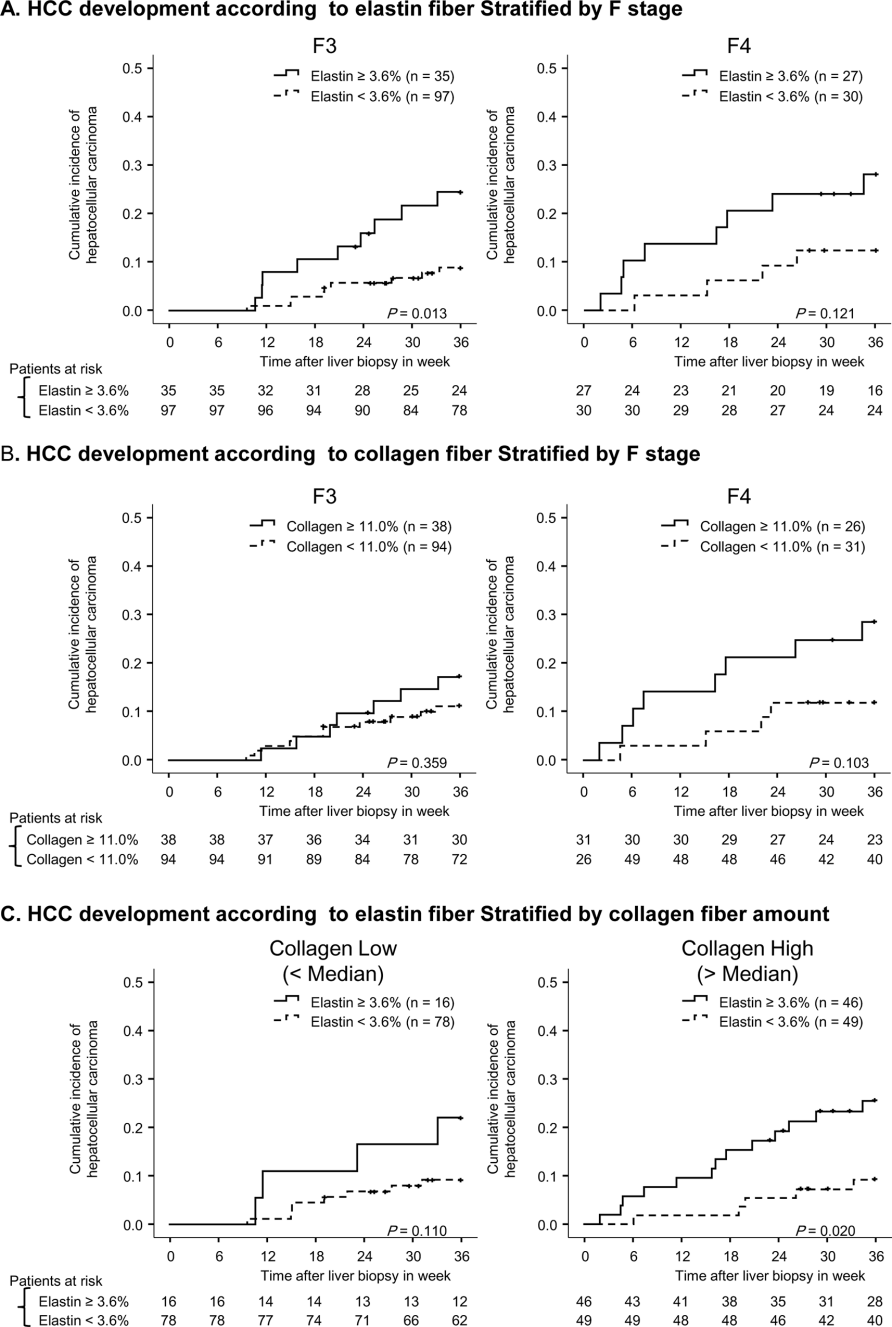
Cumulative incidence of HCC according to fiber content after stratification. Panel (A) compares the cumulative incidence of HCC in patients with high elastin (solid line) and low elastin (broken line) after stratification by METAVIR F stage. Panel (B) compares the cumulative incidence of HCC in patients with high collagen (solid line) and low collagen (broken line) after stratification by METAVIR F stage. Vertical lines indicate censored cases. Panel (C) shows the cumulative incidence of HCC after stratification by collagen. Low and high collagen were separated by median value.

In total, 30 (16%) patients developed HCC within the period of follow-up. The cumulative incidence of HCC was 5%, 12%, and 16% at one, two, and three years, respectively. To predict the development of HCC using receiver operating characteristic curve, 11.0% and 3.6% were defined as cut off values for collagen proportional area and elastin proportional area, respectively. Patients with higher collagen showed a significantly higher incidence of HCC (*p* = 0.05). Similarly, patients with higher elastin showed a significantly higher incidence of HCC (*p* = 0.002) (Fig 4). Patients with both high elastin and high collagen had higher risk of HCC compared to patients with either high elastin or high collagen. Therefore, the assessment of both collagen and elastin had additional value on that of collagen or elastin alone (Fig 4C). After stratification by F stage, elastin was a significant predictor for the development of HCC among F3 patients (*p* = 0.01), showing a similar tendency among F4 patients (*p* = 0.12) (Fig 5A). In addition, F4 patients with higher collagen content showed higher incidences of HCC (*p* = 0.10). However, among F3 patients no significant difference between patients with high and low collagen content was observed (*p* = 0.36) (Fig 5B). Furthermore, even after stratification by collagen proportional area, patients with higher elastin correlated a higher incidence of HCC (low collagen, *p* = 0.11; high collagen, *p* = 0.02). Therefore, collagen and elastin fibers independently correlate with HCC development.

### Patients who have higher elastin fibers

In total, 62 out of 189 showed higher elastin proportional area (>3.6%). Patients who have higher elastin fibers showed lower platelet count (10.8 vs. 12.7, p = 0.002), higher INR (1.05 vs. 1.02, p = 0.02), and higher collagen proportional area (12.1% vs. 8.9%, p < 0.001). METAVIR F4 stage was significantly frequent in patients with higher elastin fibers (44% vs. 24%, p = 0.001). No differences were observed in sex, transaminases, bilirubin, albumin, glucose, and alpha-fetoprotein between patients with higher and lower elastin fibers.

### Factors associated with the development of HCC

Table 2 shows factors associated with the development of HCC. In univariate analysis, age, platelet, PT-INR, FIB-4 index, collagen fiber, and elastin fiber were significant risk factors for HCC. In multivariate analysis with variables which showed p < 0.1 in univariate analysis using backward elimination method, elastin fiber as well as age, FIB-4 index, and PT-INR were significant independent factors for the development of HCC.

**Table 2:** Factors associated with HCC development.

Variables	Univariate			Mulivariate		
	OR	95% CI	*p*	OR	95% CI	*p*
Age (> 70)	2.74	(1.32–5.70)	0.007	2.54	(1.21–5.31)	0.01
Gender (Male)	1.66	(0.81–3.41)	0.16			
Fibrosis (F4)	1.65	(0.79–3.42)	0.18			
AST (> 38 IU/L)	1.45	(0.68–3.09)	0.34			
ALT (> 43 IU/L)	0.65	(0.32–1.32)	0.23			
Bilirubin (> 1.0 mg/dl)	1.44	(0.61–3.37)	0.40			
Platelet (≤ 10.6 × 10^4^ /μL)	2.23	(1.09–4.57)	0.03			
Albumin (≤ 4.0 g/dL)	2.33	(0.89–6.10)	0.08			
GGT (> 80 IU/L)	0.67	(0.26–1.77)	0.42			
Glucose (> 120 mg/dL)	1.37	(0.64–2.92)	0.42			
Cholesterol (≤ 160 mg/dL)	1.93	(0.90–4.11)	0.09			
Triglyceride (≤ 110 mg/dL)	1.36	(0.63–2.90)	0.43			
PT-INR (> 1.07)	3.36	(1.64–6.88)	<0.001	2.42	(1.17–4.98)	0.02
APRI (> 1.75)	1.95	(0.95–4.01)	0.07			
FIB-4 index (> 3.5)	6.84	(1.63–28.7)	0.009	4.38	(1.02–18.8)	0.05
HCV genotype (1b)	2.26	(0.29–17.4)	0.43			
Collagen proportional area (>11.0%)	2.04	(1.00–4.17)	0.05			
Elastin proportional area (>3.6%)	3.00	(1.45–6.17)	0.003	2.45	(1.18–5.09)	0.02

Abbreviations: AST, aspartate aminotransferase; ALT, alanine aminotransferase; GGT, gamma glutamyl transferase; APRI, AST to Platelet Ratio Index; CI, confidence interval.

## Discussion

Cirrhosis is a leading cause of HCV-related HCC, and therefore, liver fibrosis has been considered to play a main role in hepatocarcinogenesis [17]. To date, the degree of fibrosis is diagnosed by staging systems such as METAVIR staging. This staging system consists of analysis of morphological change such as bridging fibrosis, whereas quantification of fiber is not a criterion for diagnosis. Therefore, each patient in the same fibrosis stage may have a different fiber amount. Recently, several investigators mentioned that quantification of collagen fibers can predict the outcomes of cirrhosis patients. Using more precise quantification with computational method, the present study revealed the variability of fiber amounts even among patients with similar stages of fibrosis. We also clarified that patients who had higher amounts of collagen fibers were at higher risk of developing HCC than patients with lower amount of collagen fibers within the same fibrosis stage. Thus, measuring fibers has an additive value on the fiber staging system because of its quantitativity. In fact, the present study revealed the patients who diagnosed as the same stage of conventional staging system could be stratified by the amount of fibers.

The present study also demonstrates that patients with higher amounts of elastin fiber had a significantly higher risk of HCC development. A previous study reported that the amount of elastic fiber increased with fibrosis progression in nonalcoholic steatohepatitis patients [18]. Our study clarified that this increase could also be seen in chronic hepatitis C patients. Furthermore, the present study revealed the relationship between elastin fiber and the development of HCC. Even after the stratification by F stage or collagen amount, patients with higher amounts of elastin fiber had higher risk of developing HCC within 3 years. Elastin fiber appears to be a significant predictor for the development of HCC especially at the F3 stage. In general practice, for patients with moderately advanced fibrosis, the risk of HCC was difficult to evaluate. Therefore, elastin fiber quantification will likely be a novel predictor for near future HCC development for those patients. On the other hand, at the F4 stage, patients with higher amounts of collagen fibers showed a tendency of having a higher incidence of HCC. Thus, collagen fiber quantification will be useful for identifying high risk patients with F4 stage fibrosis. The present study indicated that at least in patients with advanced fibrosis, elastin fiber in addition to collagen fiber quantification is useful for further risk classification of the development of HCC. For patients with non-advanced fibrosis, the usefulness of fiber quantification especially for elastin fiber needs to be evaluated with further investigation.

In chronic liver injury, elastin fiber deposition is increased along with other extra cellular matrix (ECM), such as collagen types I and IV and hyaluronan [19]. TGF-beta, which is over-expressed in activated stellate cells (HSC) [20], acts as a positive regulator for ECM production [21–23]. In addition to fibrogenesis, the development of HCC is induced by TGF-beta through the Smad3 signaling pathway [24, 25]. Furthermore, TGF-beta is also a promoter of tumor progression and a target for therapy [26, 27]. From that point of view, HCC development and fiber deposition are both results of such cytokine expression, and fiber amount will be a pathological quantifiable biomarker of responsible cytokines. However this mechanism is not only true about elastin fiber deposition, but also collagen fiber deposition. As for now, there is little that have mentioned about elastin particular synthesis or degradation. A recent report clarified that in mice elastin fibers are degraded by MMP-12 [28], which might play a key role of individual difference. In the clinical setting, European investigators reported a retrospective study that supports the linkage between elastin fiber content and hepatic decompensation, including the development of HCC [29]. Another interesting report on elastin from Germany [30] revealed elastin-based molecular MRI as a potential diagnostic tool in animals. This result may lead to future noninvasive imaging techniques for assessing liver elastin accumulation.

We also confirmed the usefulness of automated fiber quantification with high-resolution whole-slide image analysis. Whole slide imaging is known to be useful for education, tele-consultation regarding pathology, and research. Among the many fields in which whole slide imaging is used, one of the unique techniques is computerized quantification, increasingly being applied to several organs [13]. To quantify structures occupying small amounts of space, this method appears indispensable.

The limitation of the present study is that we evaluated liver biopsy specimens which comprise only a small amount of the entire liver. Since liver fibrosis sometimes differs by location, we should accept that a liver biopsy specimen is only a representative of the whole liver. However, in chronic hepatitis C patients, the fibrosis pattern is of the small nodular type. Therefore, error due to the use of biopsy specimens is unlikely to occur contrary to the situation represented by large nodular cirrhotic disease such as hepatitis B. The other limitation is that we only studied non-SVR patients. As the risk of HCC is not negligible for cirrhotic patients with SVR, future large-scale study enrolled both SVR and non-SVR patients is demanded. Last, due to the retrospective design of the study, some important risks including HCV RNA and HOMA-IR levels for HCC were lacking. Future prospective study may be indicated for comprehensively surveying all the potential risks of HCV-associated HCC.

In conclusion, hepatic fiber quantification is predictive for the HCC development. Elastin fiber accumulation is especially associated with the development of HCC independently of collagen fiber content. Further basic and clinical studies are required to reveal the mechanism of this phenomenon and the possibility of reversibility.

## Supporting Information

S1 Fig The flow diagram of automated quantification of fibers. Automated quantification of fibers consists of i) acquiring of WSI of each specimen, ii) automatic color calibration, iii) color classification, and iv) outputting median value of fibers obtained from each 1mm^2^.
(TIF)

## Abbreviations

HCC: hepatocellular carcinoma
SVR: sustained virological response
HCV: hepatitis C virus
IFN: interferon
WSI: whole slide image
SD: standard deviation
