# Correction: Elastin Fiber Accumulation in Liver Correlates with the Development of Hepatocellular Carcinoma

**DOI:** 10.1371/journal.pone.0160133

**Published:** 2016-08-15

**Authors:** Yutaka Yasui, Tokiya Abe, Masayuki Kurosaki, Mayu Higuchi, Yasuyuki Komiyama, Tsubasa Yoshida, Tsuguru Hayashi, Konomi Kuwabara, Kenta Takaura, Natsuko Nakakuki, Hitomi Takada, Nobuharu Tamaki, Shoko Suzuki, Hiroyuki Nakanishi, Kaoru Tsuchiya, Jun Itakura, Yuka Takahashi, Akinori Hashiguchi, Michiie Sakamoto, Namiki Izumi

Figs 4 and 5 are incorrect. The horizontal axis title is incorrect. The correct title is: Time after liver biopsy in month. The authors have provided corrected versions here.

**Fig 4:**
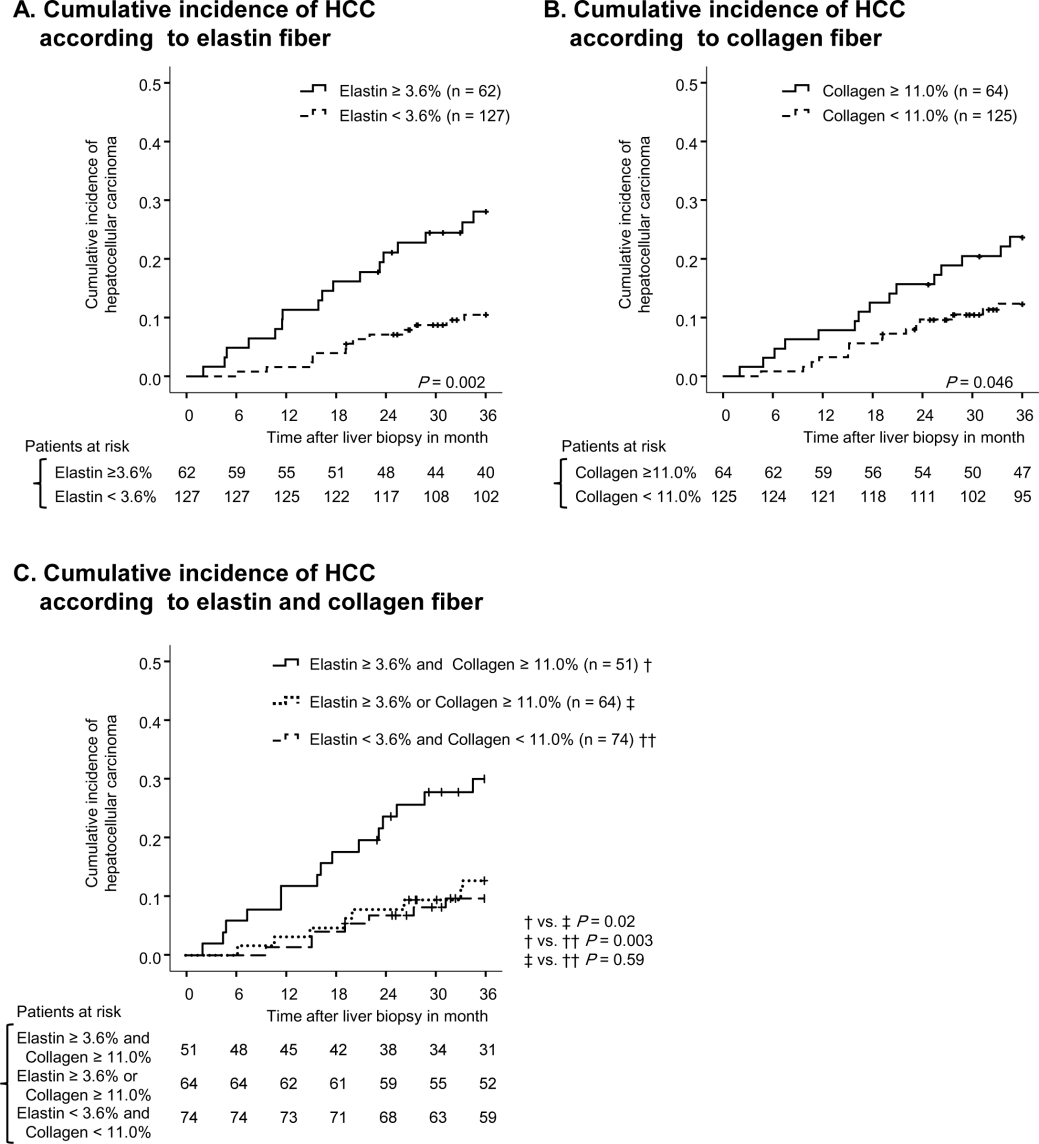
Cumulative incidence of HCC according to fiber content. On panel (A), the cumulative incidence of HCC is compared between patients with high elastin (solid line) and low elastin (broken line). On panel (B), the cumulative incidence of HCC is compared between patients with high collagen (solid line) and low collagen (broken line). Vertical lines indicate censored cases. On panel (C), the cumulative incidence of HCC is compared in three groups divided by collagen and elastin content.

**Fig 5:**
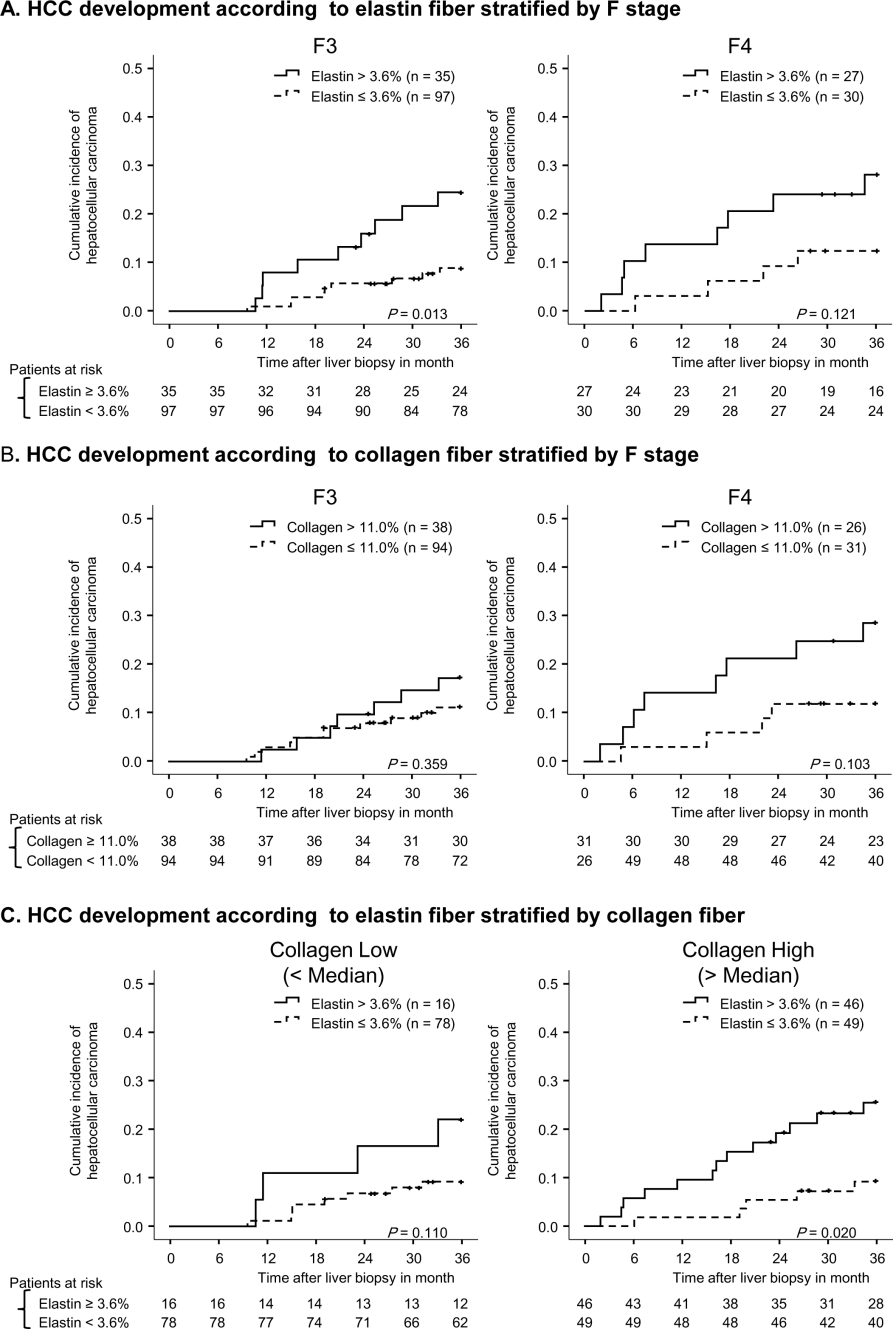
Cumulative incidence of HCC according to fiber content after stratification. Panel (A) compares the cumulative incidence of HCC in patients with high elastin (solid line) and low elastin (broken line) after stratification by METAVIR F stage. Panel (B) compares the cumulative incidence of HCC in patients with high collagen (solid line) and low collagen (broken line) after stratification by METAVIR F stage. Vertical lines indicate censored cases. Panel (C) shows the cumulative incidence of HCC after stratification by collagen. Low and high collagen were separated by median value.
