# Supplementary figures and images for: Elastin Fiber Accumulation in Liver Correlates with the Development of Hepatocellular Carcinoma
Source: PLoS One. 2016 Apr 29;11(4):e0154558. doi: 10.1371/journal.pone.0154558 (PMC4851385; doi:10.1371/journal.pone.0154558)

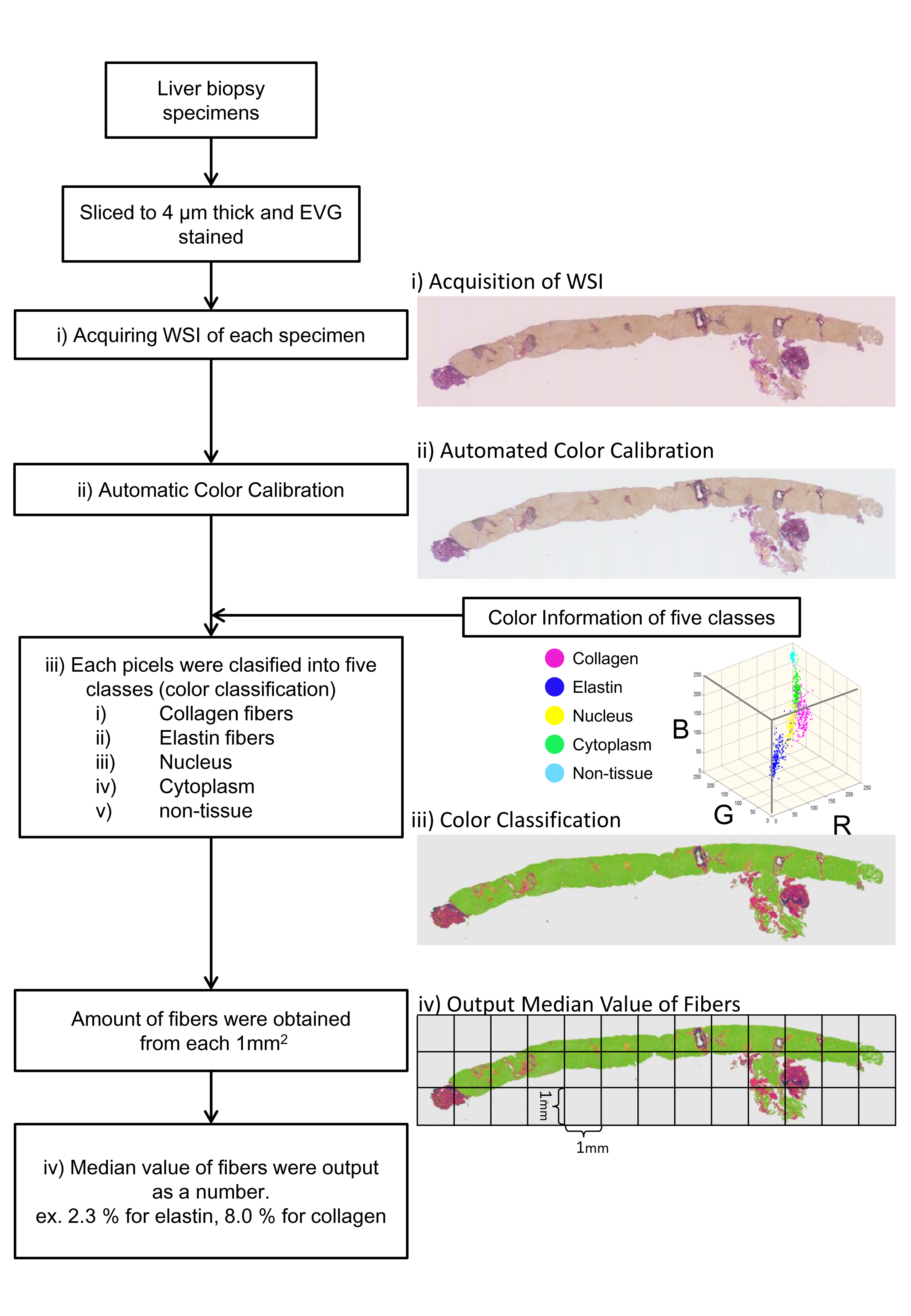

Supplement: S1 Fig — Automated quantification of fibers consists of i) acquiring of WSI of each specimen, ii) automatic color calibration, iii) color classification, and iv) outputting median value of fibers obtained from each 1mm2. (TIF) [file pone.0154558.s001.tif]
